# Distinct Patterns of Brain Atrophy associated with Mild Behavioral Impairment in Cognitively Normal Elderly Adults

**DOI:** 10.7150/ijms.60810

**Published:** 2021-06-11

**Authors:** Jun Shu, Qiang Qiang, Yuning Yan, Yang Wen, Yiqing Ren, Wenshi Wei, Li Zhang

**Affiliations:** 1 Department of Neurology, Huadong Hospital affiliated to Fudan University, No. 221, West Yan An Road, Shanghai, China.; 2 Department of Neurology, The Third People's Hospital of Chengdu, China.

**Keywords:** mild behavioral impairment, magnetic resonance imaging, voxel-based morphometry

## Abstract

A cross-sectional study was conducted to evaluate patterns of gray matter changes in cognitively normal elderly adults with mild behavioral impairment (MBI). Sixteen MBI patients and 18 healthy controls were selected. All the participants underwent a neuropsychological assessment battery, including the Mini-mental State Examination (MMSE), Geriatric Depression Scale (GDS), Self-rating Anxiety Scale (SAS), and Chinese version of the mild behavioral impairment-checklist scale (MBI-C), and magnetic resonance imaging (MRI) scans. Imaging data was analyzed based on voxel-based morphometry (VBM). There was no significant difference in age, gender, MMSE score, total intracranial volume, white matter hyperdensity, gray matter volume, white matter volume between the two groups (*p* > 0.05). MBI group had shorter education years and higher MBI-C score, GDS and SAS scores than the normal control group (*p* < 0.05). For neuroimaging analysis, compared to the normal control group, the MBI group showed decreased volume in the left brainstem, right temporal transverse gyrus, left superior temporal gyrus, left inferior temporal gyrus, left middle temporal gyrus, right occipital pole, right thalamus, left precentral gyrus and left middle frontal gyrus(uncorrected *p* < 0.001). The grey matter regions correlated with the MBI-C score included the left postcentral gyrus, right exterior cerebellum, and left superior frontal gyrus. This suggests a link between MBI and decreased grey matter volume in cognitively normal elderly adults. Atrophy in the left frontal cortex and right thalamus in MBI patients is in line with frontal-subcortical circuit deficits, which have been linked to neuropsychiatric symptoms (NPS) in dementia. These initial results imply that MBI might be an early harbinger for subsequent cognitive decline and dementia.

## Introduction

Neuropsychiatric symptoms (NPS) are standard non-cognitive features of dementia. NPS in dementia can occur at any stage in the disease and are associated with accelerating the rate of cognitive decline 1 and more significant caregiver burden 2. There is an increasing recognition that NPS are also present in elderly adults with normal cognitive function, known as mild behavioral impairment (MBI) 3. A growing body of evidence shows that MBI in cognitively normal older adults can accelerate the decline of their cognitive function and progression to mild cognitive impairment (MCI) and dementia alone or along with MCI 4-7. Two five-year longitudinal follow-up studies reported that about 70% of patients with MBI eventually progressed to dementia and MBI patients with MCI were more likely to develop Alzheimer's dementia (AD), whereas MBI patients without MCI were more likely to convert into frontotemporal dementia(FTD) 4, 8. Lately, some biomarkers of neurodegenerative disease including neurofilament light 9, tau 10, and amyloid 11; and genetic risk for AD 12 in cognitively normal samples were found to associate with MBI. A recent study found that MBI scores were associated with global and striatal amyloid-PET signal, but not with tau-PET signal 11. Conversely, another study showed that MBI scores were associated with higher tau-PET signal in the entorhinal cortex/hippocampus 10. This discrepancy could be attributed to diverse populations. MBI in elderly adults may be considered a potentially early harbinger of cognitive decline and dementia. Thus, it is crucial to capture the MBI population at high risk for cognitive decline and dementia with the hypothesis that early identification and treatment of behavioral symptoms in neurodegenerative diseases may delay dementia onset or slow its course 3.

To characterize MBI, the MBI Checklist (MBI-C), a screening scale designed to elicit emergent NPS according to the MBI criteria, was developed as a validated and efficient MBI case ascertainment tool 13. Moreover, the previous two studies have determined cutoff points of the MBI-C in detecting MBI in individuals with MCI 14 and subjective cognitive decline (SCD) 15. Cui Y et al. 16 demonstrated that the Chinese version of MBI-C has high reliability and validity and could replace the NPI-Q for AD dementia screening in the Chinese population.

Voxel-based morphometry (VBM) is known as a neuroimaging technique for detecting local changes in brain structure. There is also evidence that VBM can assist in AD diagnosis and early identification of high-risk MCI population 17. However, no study to date has investigated the changes of brain structure in MBI patients. The purpose of this study was to determine whether there have been anatomical structural changes of the brain in cognitively normal older adults with MBI (the MBI group) compared to those without MBI (the normal control group) using the VBM technique.

In this study, we employed MBI-C to evaluate NPS and used clinical quality magnetic resonance imaging (MRI) that also could detect changes of regional brain volume reported by Nowrangi MA et al al. 18 to assess regional gray matter volume. A substantial body of literature has shown that NPS's presence in dementia is associated with the structure and function impairment of some distinct brain regions such as the insula, cingulate, prefrontal cortex, striatum, and amygdala 19-21. Therefore, we posed the hypothesis that there might be some anatomical structural changes of the brain in MBI patients. These brain regions of structural changes may be in line with those related to NPS in dementia.

## Methods

### Participants and study design

We conducted a cross-sectional study involving thirty four cognitively normal older participants aged ≥50 years from inpatient neurology at the Huadong Hospital, Shanghai, China (from August 2018 to August 2019). All participants received clinical, neurological, neuropsychological, and brain MRI examinations. Patients' clinical data (e.g., age, sex, education, hematology test) and neuroimaging data were collected. All participants met the following Mini-mental State Examination (MMSE) scores for education level: 1) middle school level and above >24 points; 2) primary school level (education years >6)>20 points; 3) illiterate >17 points. Patients with the presence of acute cerebrovascular disorders, or history of other neurological disorders (e.g., epilepsy, brain tumor, normal pressure hydrocephalus, progressive nuclear palsy, multiple sclerosis), any systemic disease that can cause mental and behavioral symptoms (e.g., vitamin B12 deficiency, hepatic encephalopathy, hypothyroidism), psychiatric illness (e.g., schizophrenia, bipolar disorder, major depression), alcohol or drug abuse or addiction, or diagnosed with Alzheimer's disease or frontotemporal dementia or Lewy body dementia and other neurodegenerative diseases were excluded. A total of 16 patients who met the ISTAART-AA MBI criteria 3 and MBI-C score >8 were included in the MBI group, and 18 patients who met MBI-C = 0 were included in the control group.

This study was approved by the ethics committee of Huadong Hospital (2019K145), and informed consent was obtained for all participants.

### Neuropsychological assessment

All the participants completed a neuropsychological assessment battery, including the MMSE, Geriatric Depression Scale (GDS), Self-rating Anxiety Scale (SAS), and Chinese version of MBI-C. The MBI-C 13, a 2-page questionnaire consisting of 34 items, was structured to be consistent with the five MBI criteria domains: (1) decreased drive/motivation: 6 questions including assessments of cognitive, behavioral and emotional apathy; (2) affective/emotional dysregulation: 6 items including low mood, anhedonia, hopelessness, and guilt, and 1 question each for worry and panic; (3) impulse dyscontrol: 12 questions assessing agitation, aggression, impulsivity, recklessness, and abnormal reward, and reinforcement; (4) social inappropriateness: 5 questions describing sensitivity, empathy, and tact; (5) abnormal thoughts/perception: 5 questions assessing suspiciousness, grandiosity, and auditory and visual hallucinations. For each item, a “yes” or “no” question is followed by a severity rating scale of 1, 2, 3, respectively mild, moderate, severe. Symptoms should be persistent for no less than six months and represent a meaningful change from baseline.

### MRI acquisition and preprocessing

High-resolution structural MRI data of the brain were acquired for each patient with MAGNETON Skyra3.0T at the Department of Radiology, Huadong Hospital, Shanghai. MRI was performed on all subjects, including horizontal T1WI and T2WI, diffusion-weighted imaging (DWI), fluid-attenuated inversion recovery (FLAIR). The scanning parameters were horizontal T1WI: repetition time/echo time (TR/TE) = 220/2.46 ms, slice thickness = 5 mm, spacing = 1.0 mm, FOV = 23.00 cm; T2WI: TR/TE = 4000/92 ms, slice thickness = 5 mm, FOV = 24.00 cm; FLAIR: TR/TE = 7000/85 ms, slice thickness = 5 mm, FOV = 23.00 cm; DWI: TR/TE = 1300/62 ms, slice thickness = 5 mm, FOV = 24.00 cm. Structural MRI images were preprocessed using the CAT12 (http://www.neuro.uni-jena.de/cat/) toolbox of the SPM12 (Statistical Parametric Mapping, V.12b; http://www.fil.ion.ucl.ac.uk/spm) running in Matlab V.R2014b (uk.mathworks.com/products/matlab/). Firstly, the primary DICOM format of T1MRI images of all subjects were converted into NIFTI format files by the DICOM import function of spm12. Secondly, the converted NIFTI images were segmented into the gray matter (GM), white matter (WM), and cerebrospinal fluid (CSF). Thirdly, the gray matter was spatially normalized and modulated in DARTEL (Diffeomorphic Anatomical Registration through Exponentiated Lie Algebra) method 22 and registered to Montreal Neurological Institute (MNI) space. The modulated and normalized but unsmoothed GM images were visually inspected for artifacts using the sample homogeneity module. Those participants whose GM volume was two or more standard deviations outside of the GM volume sample distributions center would be excluded. No participant was excluded in this study. Lastly, 8 mm full-width at half maximum (FWHM) isotropic Gaussian kernel was adopted to smooth the above preprocessed GM image. The smoothed datasets were prepared for statistical analysis.

### Statistical analysis

The demographical and clinical data analysis was completed with SPSS 23.0. All the continuous variables were shown by mean ±standard deviation, and the constituent ratio described gender. Normality tests were performed by the Shapiro-Wilk test because the sample size of our study was less than 50. Two independent sample t-test was applied when samples satisfied the standard normal distribution. Two independent samples non-parametric test (Mann-Whitney U test) was done when samples did not meet a standard normal distribution. Fisher's exact test was conducted to compare the gender difference between the two groups because of the small sample size (< 40).

For image analysis, the smoothed datasets from the above preprocessed GM image were analyzed using *a* linear regression analysis to compare the differences of grey matter volume between the MBI group and the control group and to explore gray matter changes correlated to the MBI-C score in the MBI group. Age, years of education, sex, and total intracranial volume (TIV) were included as covariates. Significant effects were determined at the threshold of uncorrected *p* < 0.001 and with cluster size above 20 voxels. The image analysis was using SPM12 software.

## Results

### Clinical characteristics of patients

The demographic characteristics of the MBI group and normal control group are displayed in Table 1. There were no significant differences between the MBI group and the controls concerning age, gender, MMSE score, TIV, white matter hyperdensity, gray matter volume, white matter volume (*p* > 0.05). Significant group differences were founded in education years, MBI-C score, GDS, and SAS scores between the two groups (*p* < 0.05). Compared to the normal control group, the MBI group had shorter education years and higher MBI-C score, GDS, and SAS scores.

### Comparison of grey matter volume between the MBI group and control group

**Figure 1:**
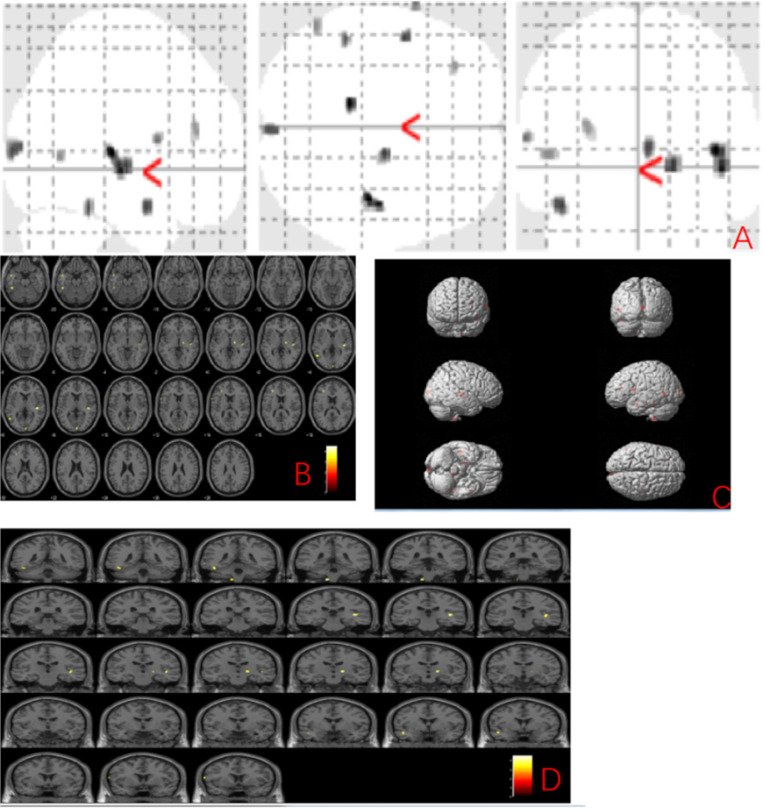
** Anatomic location of brain regions showing significant gray matter volume decrease in the mild behavioral impairment group compared to the control group.** Upper panel: Maximum density transparency graphs (A), where gray matter volume reduction was labeled with grey color. Lower panel: Horizontal (B), coronal (D) brain graphs, where gray matter volume reduction was labeled with yellow color; 3-D brain surface rendering graphs (C), where gray matter volume reduction was labeled with red color.

The MBI group showed gray matter atrophy in the left brainstem, right temporal transverse gyrus, left superior temporal gyrus, left inferior temporal gyrus, left middle temporal gyrus, right occipital pole, right thalamus, left precentral gyrus and left middle frontal gyrus (uncorrected *p* < 0.001) compared to the normal control group (Fig. 1 and Table 2).

### Grey matter volume changes related to MBI-C score

**Figure 2:**
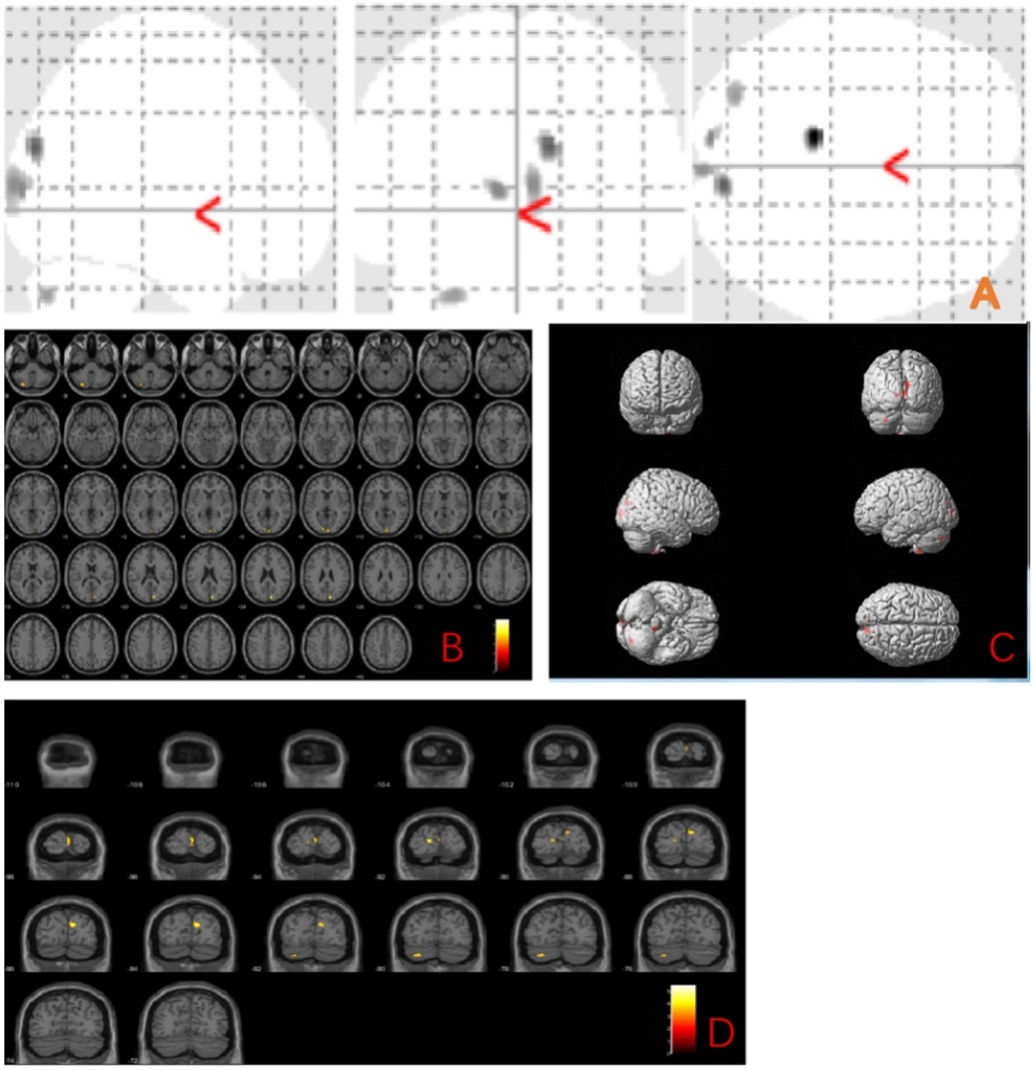
** Anatomic location of brain regions showing significant correlations between gray matter atrophy and MBI-C score.** Upper panel: Maximum density transparency graphs (A), where gray matter volume reduction was labeled with grey color. Lower panel: Horizontal (B), coronal (D) brain graphs, where gray matter volume reduction was labeled with yellow color; 3-D brain surface rendering graphs (C), where gray matter volume reduction was labeled with red color.

We further explored the gray matter volume changes correlated to the MBI-C score and found the grey matter regions correlated with the MBI-C score included left postcentral gyrus, right exterior cerebellum, and left superior frontal gyrus (uncorrected *p* < 0.001) (Fig. 2 and Table 3).

## Discussion

To our knowledge, this was the first study to investigate patterns of gray matter changes in MBI patients based on VBM. In addition, the MBI-C was used to capture MBI in our study, which is specifically designed for assessing MBI 13. The MBI-C is a more precise approach tool than the NPI or other non-specific instruments. This study showed that the MBI group had a shorter education year and higher MBI-C score, GDS and SAS scores than the normal control group. There was no difference between the MBI group and the normal control on age, gender, MMSE score, TIV, white matter hyperdensity, gray matter volume, and white matter volume. For neuroimaging analyses, we observed that MBI patients had significant decreased gray matter volume in the left brainstem, bilateral temporal cortex (mainly on the left), right occipital pole, right thalamus, left frontal cortex (uncorrected *p* < 0.001) compared to the normal control group after adjusting for TIV, age, gender, and years of education. The finding indicates associations between MBI and anatomical structural changes of the brain, which might provide insight into the anatomical basis of MBI in older adults.

The frontal cortex and thalamus are part of the frontal-subcortical circuit consisting of the frontal cortex, striatum, globus pallidus, substantia nigra, and thalamus. The circuit originates in the frontal cortex and sequentially projects to the striatum; to the globus pallidus and substantia nigra; then to the specific thalamic nuclei, and finally back to the frontal cortex and integrity of its structure and function can promote organisms to interact adaptively with their environment 23. This study showed decreased gray matter volume in the left frontal cortex and right thalamus in MBI patients, indicating that the presence of MBI in older adults may be related to the structural integrity impairment of the frontal-subcortical circuit, which was in line with the view that NPS in dementia is closely linked to impaired structural or functional integrity of frontal-subcortical circuits 24. Prior studies have consistently reported disturbance of frontal-subcortical circuits in dementia with NPS 19, 21. AD patients with apathy symptoms had significantly more significant cortical thinning in the left lateral orbitofrontal cortex (OFC), left superior and ventrolateral frontal regions than those without apathy symptoms 25. A functional MRI study revealed that MCI patients with greater affective symptoms were related to the decreased connectivity in the frontal-parietal control networks. Our findings, together with prior results, provide new evidence for a continuum between MBI and dementia. Multimodality MRI such as diffusion tensor imaging (DTI) and resting-state functional MRI (fMRI), should be applied to test the hypothesis in the future.

Our study also found gray matter structure changes of MBI patients mainly in the left temporal lobe, which, on the one hand, implied that MBI patients were in an at-risk state for incident cognitive decline and dementia, and on the other hand, supported the association of temporal lobe with NPS. The temporal lobe, especially the medial temporal lobe, is closely related to memory function 26. There is widespread agreement that the medial temporal lobe atrophy is a core bio-marker of early AD and is associated with cognitive decline and disease progression in dementia 27, 28. Patients with amnestic MCI, who converted to AD, showed bilateral gray matter loss in the medial and inferior temporal lobe at baseline compared to those who did not progress to AD 29. A recent study reported that amnestic MCI and other MCI subtypes such as dysnomic/amnestic MCI and mixed MCI all showed medial temporal lobe thinning compared to healthy elderly adults, while different MCI subtypes demonstrated unique longitudinal atrophy patterns, which may have paramount prognostic value for promoting the prediction of clinical course 30. Besides, there is some evidence that cortical thinning, tau accumulation and lower metabolism in the temporal lobe was associated with individual NPS such as apathy in both cognitively normal elderly (CN) and MCI 31, depressive symptoms in CN 32, 33, and anxiety symptoms in AD 34, respectively. These results indicate that MBI patients with temporal cortical atrophy should be closely monitored to determine whether such a population has a higher risk of conversion to MCI or dementia.

Decreased gray matter volume in the left brainstem of MBI patients was found compared with the normal control group. This difference remained significant after adjusting for sex, age, years of education, and TIV. The reticular structure is the core of the brain stem, which integrates all kinds of signals from the cortex and subcortical regions, and participates in regulating the human sleep-wake state, emotional behavior, temperature, and other functions 35. Impaired brainstem structural integrity may reduce attention networks and levels of excitement, leading to changes in emotional behavior. However, few studies on the correlation between NPS and brain stem and more studies are needed to further determine the relationship between them.

We further explored the brain regions associated with the MBI-C score and found that gray matter changes associated with the MBI-C score included the left central posterior gyrus, the left superior frontal gyrus, and the right lateral cerebellar gyrus, suggesting that cerebellar structural change was also closely related to NPS. The primary function of the cerebellum is balance and coordination, but there is increasing recognition on the role of the cerebellum in social cognition and emotion. Baillieux et al. 36 showed that small brain damage caused by trauma, tumor, infection, or stroke could cause clinically significant cognitive and affective disturbances. Wassink et al. 37 reported that decreased cerebellar volume was associated with negative psychotic symptoms in schizophrenia patients compared with normal controls. Our findings were in accordance with previous studies tending to support the hypothesis that the cerebellum also plays an essential role in social cognition and emotion.

There are several limitations worthy of discussion. Our study's sample size is relatively small, which may weaken the interpretation of our findings; therefore, future larger population-based cohorts are needed to confirm these results. This study was a cross-sectional study, and we could not evaluate the association between distinct longitudinal atrophy patterns of MBI patients and the disease progression, so longitudinal studies should be designed to elaborate on this issue. When analyzing image data, we did not use multiple comparisons correction. However, the threshold of *p* ≤ 0.001 (and not 0.05), as usually used in the VBM study, was set to overcome the chance factor 38.

In conclusion, this study shows that anatomical structural changes were present in the brain of MBI patients and VBM techniques can detect microstructural changes. Decreased gray matter volume of MBI patients in the left frontal cortex and right thalamus is in line with frontal-subcortical circuit deficits, which have been linked to NPS in dementia. The left temporal lobe atrophy was also founded in MBI patients, which is associated with cognitive decline and disease progression in patients with dementia. These initial results imply that MBI might be an early harbinger for subsequent cognitive decline and dementia.

## Figures and Tables

**Table 1:** Demographic data of the participants

	MBI group	Control group	P value
Total number (n)	16	18	
Gender (man/female)	6/10	10/8	0.327^a^
Age (years)	67.31±6.69	66.67±7.18	0.789^b^
Education (years)	9.31±1.54	11.6±3.12	0.025*^c^
MMSE score	28.19±1.22	28.78±0.81	0.103^b^
GDS score	10.19±3.78	2.67±1.50	0.000*^c^
SAS score	51.19±4.87	28.39±2.73	0.000*^c^
MBI-C total score	10.38±1.45	0.00±0.00	0.000*^c^
decreased drive/motivation	3.56±1.71	0.00±0.00	-
affective/emotional dysregulation	5.12±1.54	0.00±0.00	-
impulse dyscontrol	1.81±1.68	0.00±0.00	-
social inappropriateness	0.00±0.00	0.00±0.00	-
abnormal thoughts/perception	0.00±0.00	0.00±0.00	-
total intracranial volume (mm^3^)	1752.61±72.29	1753.32±54.90	0.975^b^
gray matter volume (mm^3^)	665.79±31.01	672.02±28.04	0.543^b^
white matter volume (mm^3^)	665.74±41.50	661.96±49.03	0.811^b^
white matter hyperdensity	13.64±3.70	13.91±5.39	0.866^b^

Note: ^a^ Fisher's Exact Tests; ^b^ two-sample t-test; ^c^ nonparametric test (Mann-Whitney U test). p<0.05 was considered significant, *represents significant difference. Abbreviations: MMSE, Mini-Mental State Examination; GDS: Geriatric Depression Scale; SAS: Self-rating Anxiety Scale, MBI-C: mild behavioral impairment checklist.

**Table 2:** Voxel-based morphometry differences between mild behavioral impairment group (n=16) and control group (n=18)

Cluster size (voxel)	Peak level	MNI Coordinates (mm)			T value	P uncorrected
		x	y	z		
68	left brainstem	-11	-38	-60	4.56	0.000
130	right temporal transverse gyrus	42	-24	6	4.54	0.000
65	Right Thalamus	17	-14	-2	4.23	0.000
40	left superior temporal gyrus	-45	0	-21	4.19	0.000
54	left inferior temporal gyrus	-45	-41	-21	4.16	0.000
89	right occipital pole	3	-95	8	4.14	0.000
23	left precentral gyrus	-65	8	12	3.97	0.000
56	left middle temporal gyrus	-53	-62	5	3.81	0.000
29	left middle frontal gyrus	-32	35	20	3.77	0.000

**Table 3:** Anatomic location of areas related to MBI-C score

Cluster size (voxel)	Peak-level	MNI Coordinates (mm)			F value	P uncorrected
		x	y	z		
147	left postcentral gyrus	-54	-18	39	43.56	0.000
62	right exterior cerebellum	44	-57	-26	32.36	0.000
36	left superior frontal gyrus	-14	54	32	28.26	0.000
